# Supplementary material for: Post-vaccination Streptococcus pneumoniae colonization and respiratory manifestations in children: A prospective cohort study
Source: PLoS One. 2025 Jul 22;20(7):e0328516. doi: 10.1371/journal.pone.0328516 (PMC12282912; doi:10.1371/journal.pone.0328516)
Supplement: Attachment — Submitted filename: RESPONSE TO REVIEWERS.docx [file pone.0328516.s003.docx]

The authors of this manuscript would like to thank the editor and reviewers of the journal Plos One for the excellent progress. We believe that we have made a remarkable improvement. Thanks to everyone involved. Here are the changes to the manuscript detailed in this document:

**Reviewer 1**

The specific comments:

Reviewer #1: This study aims to explore the relationship between pneumococcal vaccination, Streptococcus pneumoniae colonization, and potential respiratory complications following vaccination. While the findings offer valuable insights, certain issues related to sample selection, and methodology may influence the reliability and generalizability of the results.

Your notes were very important. Each one was answered as follows below.

Line 1: The title could be made more concise and precise: Post-Vaccination Streptococcus pneumoniae Colonization and Respiratory Manifestations in Children: A Prospective Cohort Study.

Thank you for the suggestion about the title. We accept the reviewer suggestion and the manuscript has a different title: “Post-Vaccination Streptococcus pneumoniae Colonization and Respiratory Manifestations in Children: A Prospective Cohort Study”

Line 35: The abstract should be organized into distinct subsections: background, methods, results, and conclusion.

We appreciate the suggestion, and the summary section now contains distinct subsections: Background, Methods, Results, Conclusion. It was modified as suggested.

Line 43: In the abstract section, it is sufficient to mention the number of participants who successfully completed the follow-up, without specifying the number of individuals who dropped out of the study.

It was modified as suggested.

“A total of 225 children were monitored, with different dates of entry into the study, which occurred between March 2018 and October 2019 (zero time)."

Line 61-67: The authors have effectively addressed the impact of the vaccination program on reducing mortality associated with pneumococcal respiratory infections. it would be beneficial to provide data on the incidence rates of both invasive and non-invasive pneumococcal disease among children in the study area, Brazil, or similar regions, both before and after the introduction of the vaccination program. Clarification is needed regarding whether the immunity conferred by the PCV10 and PCV13 vaccines primarily prevents the occurrence of infections or mitigates the severity of the disease.

Thank you for the excellent suggestion. Unfortunately, in Brazil, there is no surveillance for pneumonia or IPD in all regions of the country; therefore, there are no study data available that have clearly demonstrated a decrease in the incidence of IPD in the Southern region (site of the study in question). We have included bibliographic references as per the text below:

“In their study, Wahl et al. (2018) estimated that there were 294,000 pneumococcal deaths among children aged 1–59 months worldwide in 2015 and that these deaths decreased by 51% from 2000 to 2015 due to the widespread use of PCV accompanied by also a reduction in cases of the disease ^4,5^. The same study highlighted that Brazil reached one of the largest reductions by 2015, 88% of deaths from this agent after the introduction of the 10-valent pneumococcal conjugate vaccine (PCV10) in the country^4^. More recently, in a hospital-based study historical series on IPD among children in Brazil, fatalities were significantly reduced from 6.6 to 2.0 cases per 10,000 after the introduction of PCV10 in the public system^6^. In the state of Rio Grande do Sul, a decrease in the incidence of meningitis has been observed over time^7^.

Bibliographic references:

Bardach, A., Ruvinsky, S., Palermo, M. C., Alconada, T., Sandoval, M. M., Brizuela, M. E., Wierzbicki, E. R., Cantos, J., Gagetti, P., & Ciapponi, A. (2024). Invasive pneumococcal disease in Latin America and the Caribbean: Serotype distribution, disease burden, and impact of vaccination. A systematic review and meta-analysis. PLoS ONE, 19(6 June). <https://doi.org/10.1371/journal.pone.0304978>

Berezin, E. N., Jarovsky, D., Cardoso, M. R. A., & Mantese, O. C. (2020). Invasive pneumococcal disease among hospitalized children in Brazil before and after the introduction of a pneumococcal conjugate vaccine. Vaccine, 38(7), 1740–1745. <https://doi.org/10.1016/J.VACCINE.2019.12.038>

Centro Estadual de Vigilância em Saúde. (2023). INFORME EPIDMIOLÓGICO DAS MENINGITES 2018-2022.

Line 78: The author should clarify the statement, “post-conjugate vaccine introduction studies did not find a decrease in overall carriage rates in the population, but instead observed a simultaneous replacement by NVT." It should be noted that the introduction of conjugate vaccines led to serotype replacement, where non-vaccine serotypes (NVTs) became more prevalent within the population.
 Thank you for suggestion. We have included suggested bibliographic references and changed the text as follows.:

“Post-conjugate vaccine introduction studies have not found a decrease in overall carriage rates in the population, but rather replacement, where non-vaccine serotypes (NVT) have become more prevalent^13-15^.

Bibliographic reference:

Weinberger, D. M., Malley, R., & Lipsitch, M. (2011). Serotype replacement in disease after pneumococcal vaccination. *The Lancet*, *378*(9807), 1962–1973. <https://doi.org/10.1016/S0140-6736(10)62225-8/ASSET/F7618DAA-6766-491D-8BE2-40B062953AB9/MAIN.ASSETS/GR1.SML>

Line 110: The study's recruitment strategy, which relies heavily on one or two local schools, may introduce selection bias by potentially excluding non-school-attending children or students from other schools in different urban or rural areas. Clarify any potential biases resulting from the recruitment method. The study also heavily depends on data from prior research, limiting the capacity to control for all variables.
We agree with your comment, as the text described suggested that only 2 schools were accessed, but in fact the process involved 11 out of 13 schools in the community.

It was modified as suggested line 121 to 125: “Following the random draw order, 11 out of 13 school principals were contacted to ensure a randomized selection. School visits continued until the required sample size was reached, considering a loss of 10%, resulting in different entry and exit dates for each participant. Additional volunteers outside the school environment were recruited through an appeal on local radio.”

Line 128: Relying on non-standardized diagnostic criteria used by physicians for respiratory diseases may introduce variability and reduce diagnostic consistency. The authors should address the implications of using non-standardized diagnostic criteria across different physicians and its potential impact on the consistency of outcome measures. Could this variability have biased the results, leading to an underestimation or overestimation of disease incidence?

We agree with your comment that there really is a potential bias in relation to the diagnosis because it is a variable that is not fully controlled and this appears recorded in the study limitations section between lines 357 to 364 (text below). However, if on the one hand there is a potential bias, on the other hand all diagnoses were rigorously evaluated and confirmed/ruled out by a pediatrician on our team with extensive clinical and academic experience in respiratory diseases.

“It is also important to mention the lack of standardized definition of criteria for diagnoses among pediatricians and memory bias in interviews with the guardians (minimized by information verified against medical records).”

Line 143: The outcomes, particularly pneumonia, were rigorously defined using criteria from Shah et al., enhancing the study's internal validity. However, for pneumonia specifically, while chest X-rays were used to confirm cases, it remains unclear whether all children with suspected pneumonia underwent imaging. This potential inconsistency may have introduced bias. The authors should discuss whether all suspected pneumonia cases were subjected to chest X-ray confirmation, and how missing data or inconsistent confirmation might have influenced the study’s findings.

We agree with your observation that we do not have absolute control over this issue. However, in the community where the study was conducted, children often undergo radiologic evaluation when pneumonia is suspected in the pediatric clinic. That said, we cannot guarantee that all suspected cases underwent radiologic investigation. We emphasize that all diagnoses were rigorously evaluated and confirmed/ruled out by a pediatrician on our staff with solid expertise in respiratory diseases, as described in line 153 to 155 of the manuscript: "All pneumonia cases were reviewed and confirmed/ruled by our team's pediatrician (JCBS), considering the previously described criteria for case entry together with the demonstration of infiltrate or consolidation on chest X-ray”. However, this limitation is addressed in the study discussion section, line 357 to 364: “It is also important to mention the lack of standardized definition of criteria for diagnoses among pediatricians….”.

Line 146: Nasopharyngeal (NP) swabs were collected only at the initial time point, which may not fully capture the dynamics of colonization over time. A longitudinal sampling approach, involving multiple NP swabs over time, could have provided more comprehensive data on colonization persistence or clearance.

We agree with your comment that ideally monitoring involving multiple serial NP swabs would have provided greater consistency to the study, but it was not designed to monitor colonization over time.

Line 160: The sample size calculation appears well-justified; however, details regarding the actual statistical power based on the final dataset are missing. A more thorough discussion is needed to determine whether the achieved sample size was sufficient to detect the hypothesized effect size, particularly within subgroups. Additionally, consider addressing potential confounding factors in the Poisson regression analysis, such as socioeconomic status, healthcare access, or pre-existing health conditions.
In fact, we reconsidered some issues, we appreciate the suggestion and, as shown in tables 1, 2 and 4, we included the multivariate analysis adjusted for age and sex with the intention of clarifying possible confounding factors.

Table 1 - Cohort of children aged 18–59 months, colonized and non-colonized: general (sociodemographic) and vaccination characteristics, Veranópolis, Brazil — Interview database, n=225.

|  |  |  | Univariate | | | | Multivariate | | | |
| --- | --- | --- | --- | --- | --- | --- | --- | --- | --- | --- |
| VARIABLE | ***S. pneumoniae* (-)** | ***S. pneumoniae* (+)** | ***p*** | **RR** | **CI95%** | | ***p*** | **RR** | **CI95%** | |
|  | **n=80** | **n=145** |  |  |  |  |  |  |  |  |
|  | n (%) | n (%) |  |  |  | |  |  |  | |
| Age:    18 to 23 months | 16 (37.2%) | 27 (62.8%) |  | 1 |  |  |  | 1 |  |  |
| 24 to 35 months | 23 (36.5%) | 40 (63.5%) | 0,941 | 1,011 | 0,752 | 1,36 | 0,840 | 1,030 | 0,773-1,372 | |
| 36 to 47 months | 21 (33.9%) | 41 (66.1%) | 0,727 | 1,053 | 0,787 | 1,409 | 0,522 | 1,104 | 0,815-1,497 | |
| 48 to 59 months | 20 (35.1%) | 37 (64.9%) | 0,828 | 1,034 | 0,767 | 1,394 | 0,463 | 1,118 | 0,830-1,506 | |
| Sex:     Female | 46 (41.8%) | 64 (58.2%) |  | 1 |  |  |  | 1 |  |  |
| Male | 34 (29.6%) | 81 (70.4%) | 0,05 | 1,21 | 0,99 | 1,48 | 0,117 | 1,177 | 0,960-1,443 | |
| Household^a^:     2–3 people | 33 (35.1%) | 61 (64.9%) |  | 1 |  |  |  | 1 |  |  |
| 4–5 people | 40 (34.8%) | 75 (65.2%) | 0,961 | 1,005 | 0,823 | 1,227 | 0,585 | 1,061 | 0,857-1,314 | |
| 6 or more | 7 (43.8%) | 9 (56.3%) | 0,540 | 0,867 | 0,549 | 1,369 | 0,404 | 0,826 | 0,526-1,296 | |
| Children in the household^b^: 0-1 | 64 (33.9%) | 125 (66.1%) |  | 1 |  |  |  | 1 |  |  |
| 2–3 | 16 (44.4%) | 20 (55.6%) | 0,269 | 0,84 | 0,616 | 1,145 | 0,194 | 0,802 | 0,574-1,119 | |
| Shared bedroom^c^: No | 45 (34.1%) | 87 (65.9%) |  | 1 |  |  |  | 1 |  |  |
| Yes | 35 (37.6%) | 58 (62.4%) | 0,588 | 0,946 | 0,775 | 1,156 | 0,363 | 0,906 | 0,732-1,121 | |
| Smoker family member: No | 64 (35%) | 119 (65%) |  | 1 |  |  |  | 1 |  |  |
| Yes | 16 (38.1%) | 26 (61.9%) | 0,703 | 0,952 | 0,734 | 1,235 | 0,529 | 0,924 | 0,723-1,181 | |
| Smoke exposure^d^:  No | 36 (37.1%) | 61 (62.9%) |  | 1 |  |  |  | 1 |  |  |
| Yes | 44 (34.4%) | 84 (65.6%) | 0,671 | 1,044 | 0,856 | 1,272 | 0,870 | 1,017 | 0,834-1,239 | |
| School^e^: No | 2 (40%) | 3 (60%) |  | 1 |  |  |  | 1 |  |  |
| Yes | 78 (35.5%) | 142 (64.5%) | 0,843 | 1,076 | 0,522 | 2,215 | 0,729 | 1,128 | 0,570-2,234 | |
| Vaccination:  Mixed^f^ | 6 (42.9%) | 8 (57.1%) |  | 1 |  |  |  | 1 |  |  |
| PCV13^g^ | 3 (50%) | 3 (50%) | 0,776 | 0,875 | 0,349 | 2,195 | 0,796 | 0,887 | 0,358-2,199 | |
| PCV10^h^ | 71 (34.6%) | 134 (65.4%) | 0,570 | 1,144 | 0,719 | 1,820 | 0,599 | 1,132 | 0,713-1,796 | |

*S. pneumoniae* (-) = non-colonized, *S. pneumoniae* (+) = colonized. ^a^ Number of people living in the same household (including the child). ^b^ Number of children under 5 in the household (including the child). ^c^ Child shares the bedroom or bed with at least one person. ^d^ Use of fireplace or wood cookstove in the house. ^e^ School attendance between “zero time” and “end of follow-up”. ^f^ Mixed vaccination — the child received PCV10 and PCV13 doses. ^g^ Received 4 doses of PCV13. ^h^ Received 3 or 4 doses of PCV10.

Table 2 - Distribution of outcomes per clinic syndrome^a^ among children aged 18–59 months with medical care in record, Veranópolis, Brazil, n=225.

|  |  |  |  |  | Univariate | | | | Multivariate | | | |
| --- | --- | --- | --- | --- | --- | --- | --- | --- | --- | --- | --- | --- |
|  | ***S.pneumoniae* (-) n=80** | | ***S.pneumoniae* (+) n=145** | | ***p*** | **RR IC95%** | | | ***p*** | **RR IC95%** | | |
|  | n | % | n | % |  |  |  |  |  |  |  |  |
| Pneumonia | 2 | 2,50% | 5 | 3,40% | 0,695 | 1,38 | 0,27 | 6,95 | 0,714 | 1,37 | 0,25 | 7,36 |
| Sinusitis | 14 | 17,50% | 25 | 17,20% | 0,961 | 0,99 | 0,54 | 1,79 | 0,985 | 1,01 | 0,55 | 1,84 |
| AOM^b^ | 14 | 17,50% | 26 | 17,90% | 0,935 | 1,02 | 0,57 | 1,85 | 0,850 | 1,06 | 0,59 | 1,90 |
| Tonsillopharyngitis | 22 | 27,50% | 36 | 24,80% | 0,661 | 0,9 | 0,57 | 1,42 | 0,548 | 0,87 | 0,55 | 1,37 |
| ARI^c^ | 66 | 82,50% | 109 | 75,20% | 0,206 | 0,91 | 0,79 | 1,05 | 0,382 | 0,94 | 0,82 | 1,08 |
| Asthma | 2 | 2,50% | 9 | 6,20% | 0,217 | 2,48 | 0,55 | 11,2 | 0,319 | 2,22 | 0,46 | 10,72 |
| Antimicrobial^d^ | 41 | 51,30% | 75 | 51,70% | 0,946 | 1,01 | 0,77 | 1,32 | 0,972 | 1,00 | 0,77 | 1,31 |
| No medical care^e^ | 8 | 10% | 29 | 20% | 0,064 | 2,00 | 0,96 | 4,17 | 0,096 | 1,82 | 0,90 | 3,68 |

^a^ Presence of at least one episode of respiratory disease (pneumonia, sinusitis, AOM, ARI, asthma or tonsillopharyngitis) between “zero time” and “end of follow-up”. ^b^ AOM — acute otitis media. ^c^ ARI — Upper and lower respiratory tract infection — diagnosis classified in this category when record showed: cold, flu, rhinitis, allergic/viral rhinitis, respiratory virosis, conjunctivitis, influenza, viral tonsillitis, sore throat, nasopharyngitis, pharyngitis, laryngitis, turbinate hypertrophy, enlarged adenoids, tracheobronchitis, bronchitis, bronchiolitis, acute respiratory infection (ARI), head, foot, and mouth disease, erythema multiform, mononucleosis, adenovirus infection, unspecified viral infection, viral/external/serous AOM, positive COVID test. ^d^ Record showed one or more uses of antimicrobial between “zero time” and “end of follow-up”. ^e^ Children without medical care record in the period of interest.

Table 4 - Serotypes 6B (PCV10) X Colonized children and pneumonia diagnosis, Veranópolis, Brazil. Record database, n=145.

|  |  |  |  |  | Univariate | | | | Multivariate | | | |
| --- | --- | --- | --- | --- | --- | --- | --- | --- | --- | --- | --- | --- |
|  | **Pneumonia (-)** | | **Pneumonia (+)** | | ***p*** | **RR** | **CI95%** | | ***p*** | **RR** | **CI95%** | |
|  | n | % | n | % |  |  |  | |  |  |  | |
| 6B | 3 | 75,0% | 1 | 25% | **0,03** | 8,81 | 1,25 | 62,1 | 0,01 | 7,06 | 1,65 | 30,19 |
| Other serotypes^a^ | 137 | 97,2% | 4 | 2,80% |  |  |  |  |  |  |  | |

^a^Other serotypes: 3, 6A, 06C, 10A, 11A, 13, 15A, 15B, 15C, 16F, 16F/037, 19A, 22F, 23A, 23B, 28A, 34, 35B, NT.

Line 290: The text contains several small formatting errors, such as redundant citations and minor repetition (e.g., "24, 24" in multiple places).
 It was modified as suggested.

Line 307: The implications of the study for public health and vaccine policy are not emphasized enough, despite the study having potential relevance for the National Immunization Program in Brazil. A more explicit connection between the study's findings and how they could inform vaccine strategies or policies would be valuable. For example, the implications of continued circulation of serotype 6B should be further discussed in the context of vaccine development or the need for updated vaccines.
It was modified as suggested between lines 367 to 382.

“Although pneumococcal colonization in children has not been shown to increase the risk of respiratory diseases, the residual circulation of vaccine serotypes, such as 6B, warrants attention due to its association with pneumonia. This concern remains valid despite the reduced number of cases observed in this study, as the role of pneumococcus as the leading etiological agent of bacterial pneumonia in children cannot be underestimated. While this study did not directly evaluate vaccine effectiveness, the high vaccination coverage observed suggests that colonization in this context does not significantly alter the risk of disease, highlighting the impact of vaccines. However, it is crucial to monitor serotype replacement and the growing diversity of pneumococcal strains, which could undermine the benefits achieved. The findings reinforce the effectiveness of the strategies implemented by the National Immunization Program (PNI) and underscore the importance of preserving these achievements. Continuous strain analysis, epidemiological surveillance, and future studies are essential to guide adaptations in immunization policies, particularly regarding residual serotypes like 6B, which was the second most common in the pediatric population during the pre-PCV era and associated with severe infections. These efforts are vital to ensuring effective protection against pneumococcal disease, considering that, so far, vaccination is the only available tool.”

Line 315: The lack of association between colonization and respiratory outcomes is an important finding, yet the discussion does not explore potential reasons why this might be the case. The authors could elaborate on possible explanations, such as the effectiveness of the vaccines in preventing disease despite colonization, or the role of other pathogens and co-infections in respiratory disease outcomes. This would provide a more thorough interpretation of the results.

It was modified as suggested between lines 346 to 355.

“There is substantial variation between individuals in the immune response to vaccination and this occurrence may be linked to factors: intrinsic, extrinsic, environmental, behavioral and nutritional^37,38^. But three factors must be taken into consideration: first, serotype 6B is one of the least effective components of PCV (82.8%)^38,39^; second, there is a reduction in the serospecific response when colonization (6B) occurs before the administration of the first dose of PCV ^21,38,40^; third, natural immunity may only be achieved after many episodes of colonization or infection^38,40^. An understanding of all these factors and their impacts offers ways to improve vaccine immunogenicity and efficacy^37^.”

Bibliography:

Zimmermann, P., & Curtis, N. (2019). Factors that influence the immune response to vaccination. *Clinical Microbiology Reviews*, *32*(2). <https://doi.org/10.1128/CMR.00084-18/FORMAT/EPUB>

Line 350: The authors briefly mention the impact of the COVID-19 pandemic on the study, but this section could be expanded.

It was modified as suggested between lines 359 to 364.

“The interruption of school activities imposed by social isolation during the SARS-CoV-2 pandemic may have influenced children's exposure to pathogens and the incidence of respiratory infections; however, while the impact of social isolation on *S. pneumoniae* colonization remains underexplored, studies have observed circulation of pneumococci belonging to NVT among patients suspected of having COVID-19 during the pandemic's first year^41,42^.”

Bibliography:

Primon-Barros, M., Varela, F. H., Polese-Bonatto, M., Sartor, I. T. S., Azevedo, T. R., de David, C. N., Tonini, M. L., Stein, R. T., Scotta, M. C., & Dias, C. A. G. (2024). High prevalence of 19A pneumococcal serotype carriage during the COVID-19 pandemic in Brazil. *Brazilian Journal of Infectious Diseases*, *28*(6). <https://doi.org/10.1016/j.bjid.2024.104467>

**Reviewer 2**

COMMENTS
General
Verardo *et al* assessed the effect of S. pneumoniae colonization on risk of respiratory diseases among children aged 18-59 months who are fully vaccinated with PCV. The study is important and could contribute to knowledge in the area. Generally, it is well written but would require proofreading to fix minor language errors, as well as clarification of some technical issues.

We appreciate your comments and apologize for the language errors that have been corrected throughout the text. Your notes were very important. Each of them has been answered as follows.

Abstract
1.Lines 48-49” and the presence of only one PCV10 vaccine serotype, 6B (2.8% of colonized)”: Was it expected that more vaccine serotypes will be colonized among the study participants who have been vaccinated? Please, clarify

It was modified as suggested lines, 48 to 49: “A high colonization rate (64.4%) was observed, with only 2.8% of carriers having a PCV10 vaccine serotype, specifically 6B, as expected”.

2. Lines 51-52 “Restriction was only observed in cases of serotype 6B colonization….”: This statement is not clear. Does “restriction” mean “exception”?
 It was modified as suggested.

Introduction

1. Lines 63-64: What accounted for the decreased mortality between 2000 and 2015?

It was modified as suggest.

“In their study, Wahl et al., 2018, estimated that 294,000 pneumococcal deaths occurred among children aged 1 to 59 months worldwide in 2015, and that these deaths decreased by 51% from 2000 to 2015 due largely to the widespread use of PCV accompanied by a reduction in cases of the disease^4^.”

2. Lines 65-67: Within which period was the 88% reduction? What about the 51% reduction? These statements appear confusing, kindly reconcilie

It was modified as suggest: “The same study highlighted that Brazil reached one of the largest reductions by 2015, 88% of deaths from this agent after the introduction of the 10-valent pneumococcal conjugate vaccine (PCV10) in the country^4^.”

Methods and material

1.Study design
a. Line 93 -95: It is not clear which period the study was undertaken

It was modified as suggest: “This was a prospective, double-blind, follow-up, observational cohort study with children aged 18 to 59 months undertaken in Veranópolis-RS, Brazil from Feb/2018 to Oct/2019. Each child was followed up for up to one year after his/hers entry into the study (Feb/2019 to Oct 2020).”

b. Lines 93-100: Separate the study design from the profile of the study areas. Improve the profile of the study area by include access to healthcare i.e. number of healthcare facilities; top causes of morbidity and mortality among children 18-59 months, the position of S. pneumoniae-associated respiratory infections on the log of top causes of morbidity and mortality; map of the study area etc.
The position of respiratory infections associated with *S. pneumoniae* in the registry of the main causes of morbidity and mortality and map of the study area: these are excellent suggestions and would contribute to the description of the population, unfortunately we do not have access to these data.

It was modified as suggest lines 101-107:

“**Study design**

This was a prospective, double-blind, follow-up, observational cohort study with children aged 18 to 59 months undertaken in Veranópolis-RS, Brazil from Feb/2018 to Oct/2019. Each child was followed up for up to one year after their entry into the study (Feb/2019 to Oct 2020).

**Overview of the study area**

Profile of the study area

This town had an estimated population of 26,533 (87% urban) people in 2020, of which 781 were children aged 18–59 months. Its 0.773 Human Development Index (HDI), U$ 15,400.00 Gross Domestic Product (GDP) (IBGE, 2018), and 75.3-year-old life expectancy at birth (IBGE, 2000)^18^. The care network has 15 pediatric health services (7 public, including 1 hospital, and 8 private). In the period 2018 to 2022 there was 1 death in the age group of 1-4 years in the city (coefficient 3.8/100,000) show that this city has good lifestyle indicators.

2.Recruitment
a.Line 103: Consider substituting “10-valent conjugate vaccine” with PCV10 for consistency
It was modified as suggest.

3.Follow-up
 a. Line 119: Change “closed questions” to “close-ended questions”

It was modified as suggest.

b. Line 123: What is the difference between “SimPCV13” and PCV13? Please ensure consistency

It was modified as suggest. We apologize for the typo.

Results
 1. Line 185: The period of follow up (March/2019 to October/2020) is more than one year. Kindly reconcile this with the respective sections e.g. title, line 186 etc.

“Among the 228 participants eligible for follow-up, there was a loss rate of 1.32% (Figure 1) yielding 225 children were monitored in 2019/2020 ("end follow-up"). Each child had unique entry and exit dates, and one year after "zero time," 216 phone interviews and 9 in-person interviews were conducted.”

2. Line 190: The text in Fig.1 is blur. Kindly replace with a better image

It was modified as suggest.

3. Line 197: Change “colonized” to “colonization”.

It was modified as suggest.

4.Lines 231 “In 51% of medical care visits for both carriers and non-carriers of S. pneumoniae, there was antimicrobial prescription (51.7%, n=75/145 versus 51.3%, n=41/80)”. This statement is not clear. 51.7% vrs 51.3% do not round up to 51%, respectively. Kindly clarify

It was modified as suggest: “On average, in 51.6% (116/225) of medical care visits for S. pneumoniae carriers and non-carriers, antimicrobials were prescribed (51.7%, n=75/145 versus 51.3%, n=41 /80).”

5. Table 2: What was the comparison group for the respective variables?
We introduced a multivariate analysis in Table 2, where all variables were compared for both carrier and non-carrier groups.

Discussion
1. Line 276 “no differences”: The were differences, except that they were not statistically significant. Kindly reconcilie.

It was modified as suggest.

2. Line 340 “hyporesponsiveness due to vaccine failure”. Vaccine failure may be inherent to the vaccine, and hypo-responsiveness may be person-related outcome. Hypo-responsiveness may not be as a result of the vaccine but biological factors of the individual including genetic and nutritional. Please revise the statement

It was modified as suggest: “There is substantial variation between individuals in the immune response to vaccination and this occurrence may be linked to factors: intrinsic, extrinsic, environmental, behavioral and nutritional^37,38^. But three factors must be taken into consideration: first, serotype 6B is one of the least effective components of PCV (82.8%)^38,39^; second, there is a reduction in the serospecific response when colonization (6B) occurs before the administration of the first dose of PCV ^21,38,40^; third, natural immunity may only be achieved after many episodes of colonization or infection^38,40^. An understanding of all these factors and their impacts offers ways to improve vaccine immunogenicity and efficacy^37^.”

Other bibliographical references included:

Zimmermann, P., & Curtis, N. (2019). Factors that influence the immune response to vaccination. Clinical Microbiology Reviews, 32(2). <https://doi.org/10.1128/CMR.00084-18/FORMAT/EPUB>

Dagan, R., Jiang, Q., Juergens, C., Trammel, J., Gruber, W. C., & Scott, D. A. (2021). Carrier-Induced Hyporesponsiveness to Pneumococcal Conjugate Vaccines: Unraveling the Influence of Serotypes, Timing, and Previous Vaccine Dose. Clinical Infectious Diseases, 72(3), 448–454. <https://doi.org/10.1093/cid/ciaa083>
