# Supplementary material for: Post-vaccination Streptococcus pneumoniae colonization and respiratory manifestations in children: A prospective cohort study
Source: PLoS One. 2025 Jul 22;20(7):e0328516. doi: 10.1371/journal.pone.0328516 (PMC12282912; doi:10.1371/journal.pone.0328516)
Supplement: Attachment — Submitted filename: REVIEWER COMMENTS.docx [file pone.0328516.s001.docx]

**REVIEWER COMMENTS**

**General**

Verardo et al assessed the effect of *S. pneumoniae* colonization on risk of respiratory diseases among children aged 18-59 months who are fully vaccinated with PCV. The study is important and could contribute to knowledge in the area. Generally, it is well written but would require proofreading to fix minor language errors, as well as clarification of some technical issues.

**Abstract**

1. Lines 48-49” and the presence of **only** one PCV10 vaccine serotype, 6B (2.8% of colonized)”: Was it expected that more vaccine serotypes will be colonized among the study participants who have been vaccinated? Please, clarify
2. Lines 51-52 “Restriction was only observed in cases of serotype 6B colonization….”: This statement is not clear. Does “restriction” mean “exception”? Please reword

**Introduction**

1. Lines 63-64: What accounted for the decreased mortality between 2000 and 2015?

2. Lines 65-67: Within which period was the 88% reduction? What about the 51% reduction? These statements appear confusing, kindly reconcile

**Methods and material**

1. Study design
   a. It is not clear which period the study was undertaken

b. Lines 93-100: Separate the study design from the profile of the study areas. Improve the profile of the study area by include access to healthcare i.e. number of healthcare facilities; top causes of morbidity and mortality among children 18-59 months, the position of S. pneumoniae-associated respiratory infections on the log of top causes of morbidity and mortality; map of the study area etc.

2. Recruitment

a. Line 103: Consider substituting “10-valent conjugate vaccine” with PCV10 for consistency

3. Follow-up
   a. Line 119: Change “closed questions” to “close-ended questions”

b. Line 123: What is the difference between “SimPCV13” and PCV13? Please ensure consistency

**Results**

1. Line 185: The period of follow up (March/2019 to October/2020) is more than one year. Kindly reconcile this with the respective sections e.g. title, line 186 etc.

2. Line 190: The text in Fig.1 is blur. Kindly replace with a better image

3. Line 197: Change “colonized” to “colonization”

4. Lines 231 “In 51% of medical care visits for both carriers and non-carriers of *S. pneumoniae*, there was antimicrobial prescription (51.7%, n=75/145 versus 51.3%, n=41/80)”. This statement is not clear. 51.7% vrs 51.3% do not round up to 51%, respectively. Kindly clarify

5. Table 2: What was the comparison group for the respective variables?

**Discussion**

1. Line 276 “no differences”: The were differences, except that they were not statistically significant. Kindly reconcile

2. Line 340 “hyporesponsiveness due to vaccine failure”. Vaccine failure may be inherent to the vaccine, and hypo-responsiveness may be person-related outcome. Hypo-responsiveness may not be as a result of the vaccine but biological factors of the individual including genetic and nutritional. Please revise the statement
